# Rapid on-site identification of hazardous organic compounds at fire scenes using person-portable gas chromatography-mass spectrometry (GC-MS)—part 1: air sampling and analysis

**DOI:** 10.1080/20961790.2019.1654205

**Published:** 2019-10-30

**Authors:** Rylee Lam, Chris Lennard, Graham Kingsland, Paul Johnstone, Andrew Symons, Laura Wythes, Jeremy Fewtrell, David O’Brien, Val Spikmans

**Affiliations:** a School of Science and Health, Western Sydney University, Penrith, Australia;; b Fire & Rescue NSW, Fire Investigation and Research Unit, Greenacre, Australia;; c Operations Capability Directorate, Fire & Rescue NSW, Greenacre, Australia;; d Environment Protection Science Branch, Office of Environment and Heritage, Lidcombe, Australia;; e Hazardous Incidents and Environmental Health Branch, New South Wales Environment Protection Authority, Sydney, Australia;; f Strategic Capability, Fire & Rescue NSW, Greenacre, Australia

**Keywords:** Forensic sciences, gas chromatography-mass spectrometry, portable GC-MS, air pollution, fire, volatile organic compounds, field analysis, needle trap

## Abstract

Recent advancements in person-portable instrumentation have resulted in the potential to provide contemporaneous results through rapid in-field analyses. These technologies can be utilised in emergency response scenarios to aid first responders in appropriate site risk assessment and management. Large metropolitan fires can pose great risk to human and environmental health due to the rapid release of hazardous compounds into the atmosphere. Understanding the release of these hazardous organics is critical in understanding their associated risks. Person-portable gas chromatography-mass spectrometry (GC-MS) was evaluated for its potential to provide rapid on-site analysis for real-time monitoring of hazardous organic compounds at fire scenes. Air sampling and analysis methods were developed for scenes of this nature. Controlled field testing demonstrated that the portable GC-MS was able to provide preliminary analytical results on the volatile organic compounds present in air samples collected from both active and extinguished fires. In-field results were confirmed using conventional laboratory-based air sampling and analysis procedures. The deployment of portable instrumentation could provide first responders with a rapid on-site assessment tool for the appropriate management of scenes, thereby ensuring environmental and human health is proactively protected and scientifically informed decisions are made for the provision of timely advice to stakeholders.

## Introduction

Research and development into person-portable instrumentation has been on the rise in recent years. Many industries are realising the benefits that field portability can offer in terms of efficiency and the provision of tactical intelligence [1–4]. The implementation of person-portable instrumentation for the sampling and analysis of environmental pollutants for human and environmental protection, for example, could greatly enhance current risk management and mitigation protocols [5].

Pollution incidents trigger an emergency response from local authorities such as Hazardous Materials (HAZMAT) units and environmental protection agencies, who are responsible for the protection of the public and the environment. The release of potentially hazardous compounds into the environment requires extensive environmental monitoring and management. For these agencies to effectively perform their duties, it is imperative that accurate, contemporaneous intelligence is acquired and communicated [4]. This includes identifying the hazardous materials released, the source of the release, and the risks that they pose to human and environmental health [6]. Intelligence of this nature can be gathered through the sampling and analysis of environmental samples [7].

The provision of timely analytical data and advice to environmental protection agencies for informed risk assessment is crucial to ensure rapid and targeted risk minimisation and management responses [6]. Analytical data and advice are currently obtained via the laboratory analysis of samples collected from the incident site. There is a significant time lapse between the initial pollution event and the subsequent reporting of laboratory results, which can delay the implementation of appropriate human and environmental protection strategies. Some instances such as slow-releasing pollutants or stationary hazards, where time is not a critical factor, may be managed appropriately through environmental sampling and analysis using traditional laboratory-based instrumentation. On the other hand, there are many emergency scenarios—where the hazards are dynamic, transient and widespread—that trigger the need for an immediate and rapid assessment of the scene [6]. The demand for timely scientific intelligence in these cases can be realised through the on-site use of person-portable instrumentation.

Fires represent a pressing threat to environmental and human health that demands rapid emergency response. The release of hazardous organic compounds into the atmosphere as a by-product of combustion not only threatens the health and safety of the surrounding environment and community, but of first responders as well. As a result, it is imperative that emergency response agencies appropriately assess and manage these risks through an understanding of the release of hazardous organics into the atmosphere [8]. Current protocols are time-consuming and complex to implement, requiring the coordination of in-field sampling and the transport of these samples back to a central laboratory for subsequent analyses. These analyses are also inherently lengthy processes requiring stable and controlled environments that are regulated by external validation criteria [9]. These requirements result in significant delays between the initial incident and the eventual reporting of scientific data and advice to relevant stakeholders. First responders are often hampered by the delayed provision of comprehensive scientific advice, which may not be available until hours or days after the initial scene attendance. As it stands, comprehensive and timely scientific advice is not currently obtainable on the ground, at the time of the event.

The introduction of person-portable instrumentation into the emergency response mandate at fire scenes can provide critical intelligence on the ground that bridges the gap between the initial release of hazardous organic materials and the reporting of confirmatory results from laboratory processes.

One person-portable instrument that could fit this application is the portable gas chromatograph - mass spectrometer (GC-MS). Laboratory-based GC-MS is commonly used for the analysis of air samples collected from fire scenes [10] and is capable of separating and identifying compounds in complex mixtures. The use of portable GC-MS instrumentation for the rapid on-site analysis of air samples at fire scenes not only provides for determining the presence of hazardous organic compounds but brings with it the potential to rapidly and continuously monitor the release of hazardous organic compounds into the atmosphere. In addition, the results are more easily confirmed using full laboratory analyses, given that the same analytical technique is used in the field and in the laboratory, and the samples can be submitted to the laboratory together with the results from the portable instrument. Portable GC-MS units are becoming more commonly available, with many offering different features and capabilities [11–13]. The choice of instrument is largely dependent on the requirements of the application at hand.

The aim of this research was to explore if a portable GC-MS is capable of providing critical information from air samples collected at fire scenes. This would aid in the timely provision of analytical results, allowing emergency response agencies to more effectively manage the risks associated with the release of toxicants into the atmosphere for the proactive protection of human and environmental health.

## Materials and methods

The performance and capabilities of a portable GC-MS were evaluated for the proposed application using a series of controlled fires where small pieces of different construction materials were burnt. An air sample was taken from the smoke plume of each burn to determine the ability of the portable GC-MS to separate, detect and identify volatile organic compounds generated from an active fire. The results were processed to determine the range of compounds that were released into the atmosphere as a result of the combustion of different materials. Air samples were also collected using an air canister to allow for comparison between field-based results and conventional air sampling and laboratory analysis procedures.

### Materials

A selection of six common household and building materials were sourced from local building and hardware stores (Table 1). Each individual material was burnt separately and in triplicate, for a total of 18 controlled fires.

**Table 1.** Six materials burnt during small-scale controlled burns and their approximate sizes.

Material	Description	Approx. size (cm)
Particle board	Particle board sheet	5.0 × 2.0 × 1.0
Melamine coated particle board	Melamine coated particle board sheet	5.0 × 2.0 × 1.0
Laminated wood	Laminated plywood sheets	5.0 × 3.0 × 2.0
Flooring underlay	Floating flooring rubber underlay	5.0 × 5.0 × 0.5
Carpet with rubber backing	Pile carpet tile with a rubber backing	4.0 × 4.0 × 0.5
Rubber mat	Rubber gym mat made from recycled tyres	5.0 × 5.0 × 1.0

### Experimental set-up

**Figure 1.**
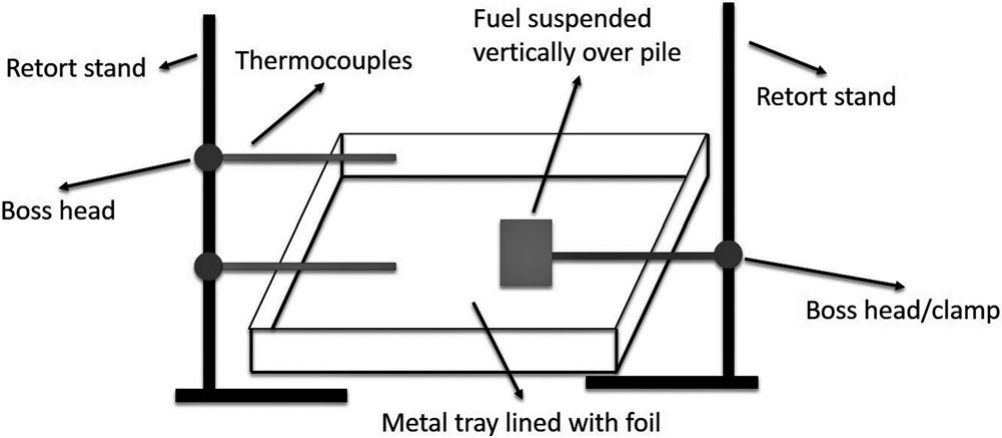
Schematic of experimental set-up.

The burns were set up over a rectangular metal tray lined with aluminium foil (Figure 1). The aluminium foil was replaced after every burn, including between triplicate burns of the same material. Two retort stands were placed adjacent to the metal tray; one retort stand was used to hold thermocouples aimed at the base and top of the fire, whilst the other retort stand held the test material vertically over a pile of paper and cardboard. Thermocouple data were used to monitor the temperature profile of each fire. The material of interest was suspended vertically to increase the surface area exposed to the flame generated by the paper/cardboard pile, promoting combustion of the material. Paper and cardboard were used to start the fire as most construction materials could not be ignited on their own. The paper/cardboard pile was lit using a match and allowed to burn fervently and ignite the material of interest. Once the material of interest was fully involved and burning, samples were collected (refer below) and water was poured over the material to extinguish the fire.

### Sampling methods

After the material of interest was engaged in active combustion, an air sample was collected from the smoke generated by the fire. Air samples were collected using a Custodion Needle Trap (NT) device (Perkin Elmer Inc., Waltham, MA, USA), consisting of a 19 ga three-phase packed-bed needle. The NT was attached to a Buck Elite vacuum pump (A.P. Buck Inc., Orlando, FL, USA) using an interface and PVC tubing [14,15]. Sorbent phases of increasing strength [14] are packed in the needle for trapping volatile and semi-volatile organic compounds (VOCs and SVOCs). The pump facilitates active sampling by drawing air through the tip of the needle and out a side hole. Analytes compatible with the sorbent phases in the packed bed are thus trapped in the needle and the remaining air matrix passes through. Active sampling increases the volume of the sample that is exposed to the sorbent bed compared to traditional solid phase microextraction (SPME) fibre sampling, thereby increasing the sensitivity of the method.

The sampling end of the NT-to-pump interface was extended with a 30-cm stainless steel tube fitted with a glass wool plug to filter out any particulate matter. The NT device was clamped in a retort stand and the stainless steel tube was positioned in the smoke plume above the flames. Air was collected at a rate of 50 mL/min for a total of 1.2 min. Accounting for the volume of the stainless steel sampling tube, this resulted in a total of 50 mL of air being passed through the NT sorbent bed. If the smoke plume moved during the collection time, the NT/stainless steel tube was moved to remain within the smoke plume. NT sampling was also performed immediately after the fire was extinguished to determine if any VOCs and/or SVOCs were being released from the fire debris at this point. Sampling rates and times for these “after extinguishment” samples were the same as for the “during fire” sampling conditions. A different stainless steel tube was used for each sample collected and the steel tubes were solvent cleaned with acetone, followed by methanol prior to use. Samples were also collected from paper and cardboard fires alone to determine the background volatile organic profile generated by the ignition materials.

Air samples were also collected using 6 L Summa air canisters (Restek Corporation, Bellefonte, PA, USA) at the same time as the “during fire” NT sample. Air canisters were cleaned and evacuated prior to sampling. Air was collected for the same length of time as for the NT sampling. The canister was fitted with an orifice that allowed 390 mL/min to be collected and a filter to prevent particulate matter from entering the canister. The canister was opened at the same time as the start of the NT sampling and closed when the NT device stopped sampling. The canister was also moved around the smoke plume if the smoke plume moved during the sampling time. The same canister was used to collect an air sample from each of the triplicate burns of the same material, combining the samples into a “summed” air sample of each material type burnt, to an approximate total air sample of 1.4 L across the three burns. A total of six air canister samples (one for each test material) were collected for subsequent laboratory analysis.

### Torion T-9 Portable GC-MS

Needle trap air samples were analysed on-site, immediately after sample collection, using a Torion T-9 Portable GC-MS (Perkin Elmer Inc.). The NT device can be directly inserted into the injection port of the T-9 for rapid, on-site analysis. Analytes trapped on the sorbent bed are thermally desorbed in the injector of the instrument. The T-9 Portable GC-MS offers features and accessories that can be efficiently utilised for environmental sampling and analysis at fire scenes.

The T-9 Portable GC-MS is a small self-contained unit weighing approximately 15 kg [11]. Due to its small size, this instrument is easily person-portable into areas not vehicle accessible and features an on-board rechargeable battery and helium cylinder for remote operation. This capability gives the T-9 flexibility to be relocated quickly in a dynamic emergency scene, as may be required in the case of an ever-changing and growing fire. With a miniaturised toroidal ion trap mass spectrometer (TMS) and low thermal mass capillary GC, the system is able to achieve rapid heating and cooling times for fast turnaround sample analysis and high sensitivity [11]. Additionally, the T-9 GC-MS has been developed with simple field sampling in mind.

Prior to conducting field analyses, the method parameters were optimised using compounds expected to be encountered during the study. The optimised parameters involved inserting the NT in the portable GC-MS and exposing the needle to 270 °C, to a 5 s desorption time. A 10:1 split ratio was applied immediately after injection and then increased to 50:1 after 10 s. The split was closed 30 s after injection. The GC temperature program consisted of a 50 °C initial temperature held for 10 s, after which the temperature was increased at a rate of 2 °C/s up until 270 °C and held for a further 60 s. After chromatographic separation, the analytes were transferred to the mass spectrometer using a transfer line temperature of 250 °C. The MS then scanned the analytes over a mass-to-charge range of 43–500 u. The total run time was 180 s.

### Air analysis GC-MS

Air canisters were analysed on a dedicated air analysis GC-MS system, comprising a Varian 3800 GC and 2000 MS (Agilent Technologies, Inc., Santa Clara, CA, USA). After sample collection, the air canisters were pressurised to 35 kPa using zero air and installed on the autosampler attached to the GC-MS. A total of 130 mL of air sample was injected into the cryotrap on the GC-MS at a flow rate of 20 mL/min. The cryotrap was kept at −150 °C, using liquid nitrogen, for the duration of the sampling. The transfer lines from the autosampler to the cryotrap were maintained at 70 °C. Once the entire sample volume was injected into the instrument, the analysis commenced by rapidly increasing the cryotrap temperature to 200 °C. The sample was injected into the GC column through a 13:1 split. The column consisted of a Restek SH-Rxi-1ms with a length of 60 m, diameter of 0.32 mm and 1.0 µm phase thickness. The GC column oven was liquid nitrogen cooled and kept at 0 °C for 7 min, after which the temperature was ramped to 160 °C at 6 °C/min. The temperature was then further ramped to 200 °C at 10 °C/min where it was held for 8.33 min. The MS transfer line temperature was 220 °C, the ion trap temperature was 160 °C and a mass range of 39–260 m/z was analysed. Data were processed using Varian Saturn MS Workstation software version 6.8 (Agilent Technologies, Inc.) with library searching conducted against the National Institute of Standards and Technology (NIST) 2011 database.

## Results and discussion

### In-field sampling and analysis

**Figure 2.**
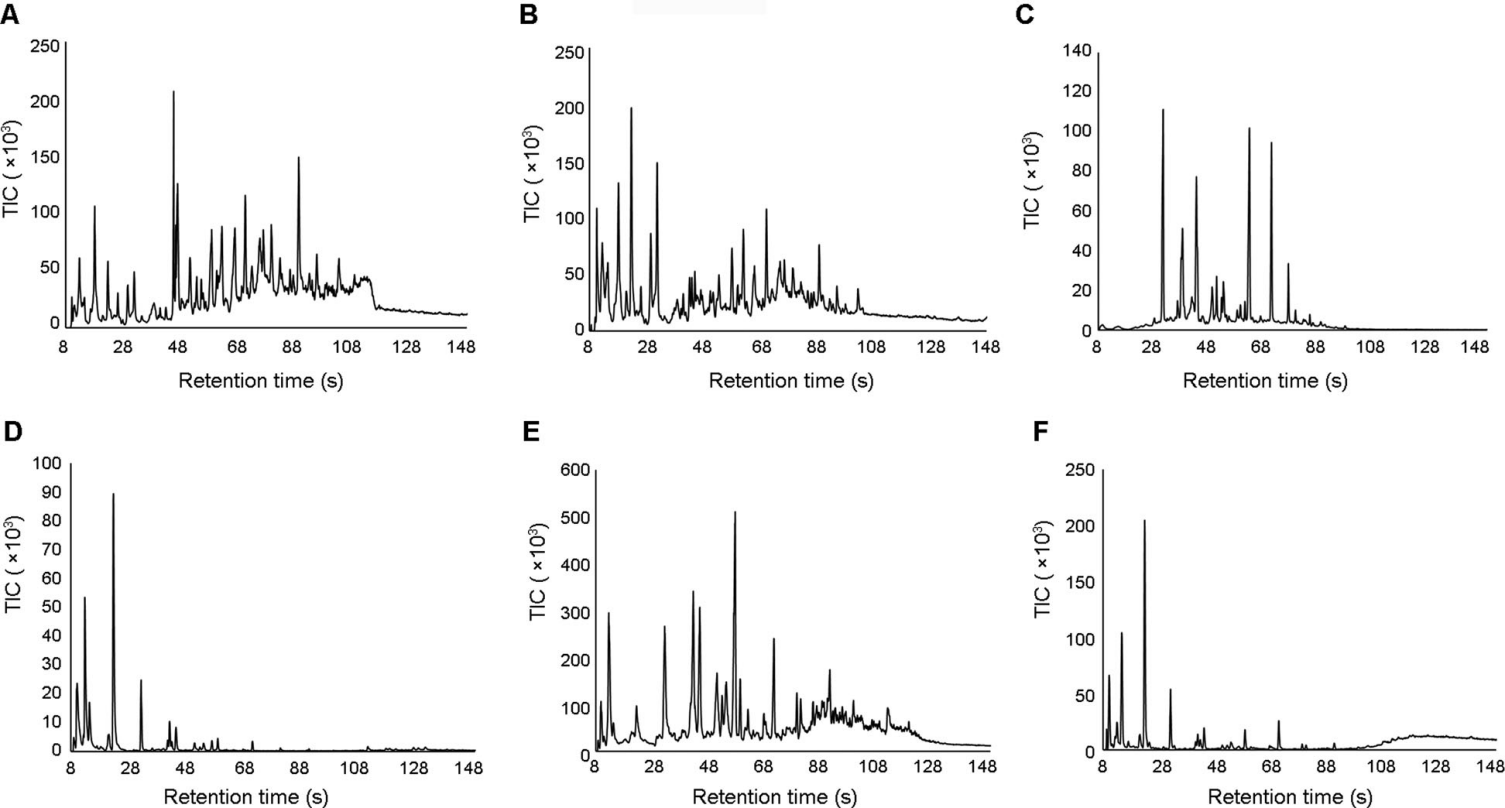
Representative chromatograms generated using the T-9 Portable GC-MS of air samples collected during fire using the NT during material burns: (A) particle board, (B) melamine coated particle board, (C) laminated wood, (D) carpet, (E) rubber and (F) underlay. TIC: total ion current.

**Figure 3.**
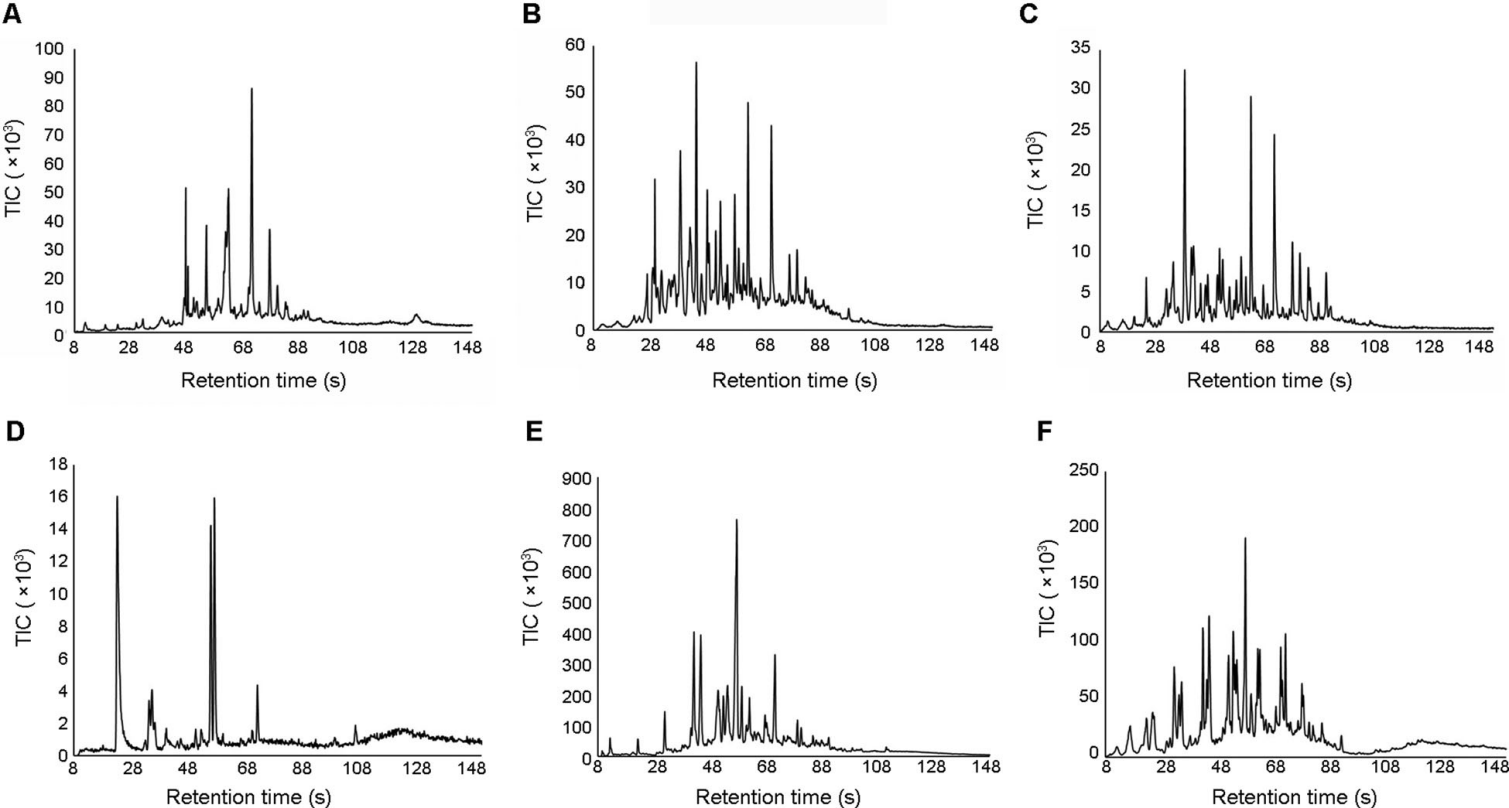
Representative chromatograms generated using the T-9 Portable GC-MS of air samples collected after extinguishment using the NT after material burns: (A) particle board, (B) melamine coated particle board, (C) laminated wood, (D) carpet, (E) rubber and (F) underlay. TIC: total ion current.

The portable GC-MS was capable of detecting and resolving compounds in the air samples collected using the NT. Figures 2 and 3 show representative chromatograms for each material burned. Despite the complex nature of air sampling, the NT and portable GC-MS were able to separate a large number of peaks within each chromatogram.

The results in Figures 2 and 3 demonstrate that the compound profiles obtained are different for the different materials burned, both during a fire and after extinguishment. Although this is to be expected, it confirms that the release of VOCs and SVOCs at fire scenes is dependent on the materials being burned. This also indicates that it is likely that different fires will produce different hazardous organic compounds and might require different risk management strategies.

### Release of VOCs and SVOCs during the fire

Of the peaks detected for the different materials burned, many were immediately identifiable using the automated data processing protocol contained within the instrument. This protocol consists of a peak deconvolution program and mass spectral matching against an on-board target library. The on-board target list consists of a library of mass spectral data produced by the instrument manufacturer. Any peaks that were not initially matched to the on-board library were deconvoluted and identified using the NIST mass spectral database [16]. This process was performed using a laptop connected to the instrument running Chromion version 1.2.0.8 software (Perkin Elmer Inc.).

Using a combination of the on-board library and additional peak identification with the NIST mass spectral database, a broad range of compounds were detected and identified by the portable GC-MS in air samples collected during the combustion of the construction materials (Table 2). Repeat compounds detected and identified across multiple burns were then added to the on-board target library for future automated on-board compound identification.

**Table 2.** Target compound list for in-field air sampling and analysis during a fire.

Compound name	PB	Melamine	LW	Carpet	Rubber	Underlay
Butanol	✔	✔			✔	✔
Dimethylpentane isomer			✔			✔
Butadiene isomer [17–19]	✔	✔		✔	✔	✔
Methylbutane isomer [20,21]		✔	✔	✔	✔	
Acrolein [22]	✔	✔	✔	✔	✔	✔
Butadiene isomer				✔	✔	✔
Acetone [18,20,22]	✔	✔		✔	✔	✔
Methylbutane isomer				✔		✔
Acrylonitrile [18,20]				✔		✔
Methylbutadiene isomer [22]	✔	✔	✔	✔	✔	✔
Furan [23]	✔	✔		✔	✔	✔
Pentenyne isomer	✔	✔	✔	✔	✔	✔
Methylpentane isomer [17]				✔	✔	
Methylpentane isomer	✔					✔
Dihydrofuran isomer	✔	✔	✔	✔		✔
Butanone isomer [22]					✔	
Hexane [17,19,21]		✔		✔		
Methylfuran isomer [22,23]	✔	✔	✔		✔	
Acetic acid [22–25]		✔				
Methyloctene isomer				✔		
Cyclohexadiene	✔	✔	✔		✔	✔
Acetic anhydride [26]	✔	✔				
Benzene [17–23,25,27–29]	✔	✔	✔	✔	✔	✔
Heptane [21]		✔			✔	✔
Ethyldimethylpentane isomer				✔		✔
Dimethylfuran isomer [22]	✔	✔	✔		✔	
Methylpyrrole isomer		✔				
Methylpyrrole isomer	✔		✔		✔	
Hexynone isomer	✔	✔	✔			
Pyridine	✔	✔			✔	
Unknown 1			✔			
Unknown 2				✔		
Toluene [17–19,21–23,25,27–31]	✔	✔	✔	✔	✔	✔
Methyleneheptane isomer [22]				✔	✔	✔
Octene isomer [22]				✔		
Unknown 3				✔		
Octene isomer				✔	✔	
Unknown 4				✔		
Furfural [22–24,26]			✔			
Furfural	✔	✔	✔	✔	✔	
Methylfuran isomer	✔	✔	✔		✔	
Methyloctane isomer				✔	✔	✔
Furanmethanol isomer [22]	✔				✔	
Furanmethanol isomer		✔	✔			
Ethylbenzene [22,23,28,29,31]	✔	✔	✔	✔	✔	✔
Unknown 5		✔				
Xylene [18,20–22,28,31]	✔		✔	✔	✔	✔
Phenylethyne [22,31]	✔	✔	✔	✔	✔	✔
Heptanone		✔		✔		✔
Styrene [17,22,23,25,27,31]	✔	✔	✔	✔	✔	✔
Xylene	✔	✔	✔	✔	✔	✔
Cyclohexanone [26]	✔	✔		✔	✔	
Unknown 6		✔				
Unknown 7	✔	✔				
Furanone isomer[26]		✔	✔			
ɑ-Pinene [22]	✔	✔	✔		✔	
Ethylhexanal isomer				✔		
Camphene [22]	✔				✔	
Propylbenzene isomer [31]					✔	
Methylfurancarboxaldehyde isomer [31]		✔	✔			
Trimethylbenzene isomer [28]	✔	✔	✔	✔	✔	
Dimethylpyrrole isomer	✔	✔	✔			
Phenol [22,23,25,27]	✔				✔	
Aniline	✔	✔	✔			
ɑ-Methylstyrene [22,28,31]	✔		✔		✔	
Trimethylbenzene isomer	✔	✔	✔		✔	
Benzonitrile [22,29,31]	✔	✔		✔	✔	
Indane [31]	✔	✔	✔		✔	✔
Dimethylpyrrole isomer	✔	✔			✔	
Benzaldehyde [22,31]				✔	✔	
Unknown 8	✔					
Trimethylbenzene isomer		✔	✔		✔	✔
Benzofuran [22,31]		✔	✔			
Methylethylheptane isomer	✔			✔		✔
Ethylhexanol isomer				✔	✔	
Unknown 9	✔					
β-Pinene	✔	✔	✔		✔	✔
Limonene [22,28]	✔	✔				
Unknown 10					✔	
Methylphenol isomer [23]			✔			
Unknown 11	✔	✔				
Indene [22,25,28,31]	✔	✔	✔	✔	✔	✔
m-Cresol	✔	✔			✔	
Acetophenone [22,31]		✔				✔
Methoxyphenol isomer [22,26,31]	✔		✔			
Methoxyphenol isomer	✔	✔				
Unknown 12	✔	✔				
Tridecane [31]				✔		
Unknown 13	✔					
Methoxymethylphenol isomer [22,23,26]		✔				
Methylindene isomer		✔		✔	✔	✔
Methoxymethylhenol isomer	✔	✔	✔			
Naphthalene [17–19,22,27,28,30–32]	✔	✔	✔	✔	✔	✔
Benzothiophene	✔	✔				✔
Ethylmethoxyphenol isomer [23]	✔	✔	✔			
Hydroxylmethylacetophenone isomer	✔	✔	✔			
Methylnaphthalene isomer [23,28,31]		✔	✔		✔	✔
Methylnaphthalene isomer			✔		✔	✔
Eugenol [24,26]	✔					
Unknown 14			✔			
Unknown 15			✔			
Biphenyl [27,31]	✔	✔			✔	✔
Tetradecane [31]		✔	✔			
Eugenol	✔	✔				
Eugenol	✔					
Acenaphthene [18,27,30,32,33]	✔		✔			✔
Acenaphthylene [18,19,22,28,30,33]	✔	✔	✔			
Ethoxymethylmethoxyphenol isomer	✔	✔	✔			
Unknown 16			✔			
Unknown 17			✔			
Fluorene [18,19,22,27,28,30,32,33]					✔	✔
Anthracene [18,19,22,30,32,33]	✔					✔

In-text citations are included for compounds previously identified within published literature searches of by-products of pyrolysis and combustion. PB: particle board; LW: laminated wood; ✔: compound detected. Note: Only the first iteration of each compound was referenced.

Compound identifications in Table 2 were made based on the similarity score and the visual comparisons between the mass spectra of the sample peaks and the instrument on-board library or the NIST database. Identifications were only provided for peaks that showed the same library match across multiple repeat analyses. Where multiple isomeric compound identifications could be made for the same peak, these were indicated in the table as the base structure, due to the difficulty associated with isomer identification without the use of standards. This included the identification of alkanes, that, without standards, cannot be identified. However, alkanes were provided in the table to provide information on the number of alkanes peaks detected in the air samples. It was not possible to identify all the compounds detected and those that could not be identified (conclusively or tentatively) have been indicated in the table as “Unknown”. The identification of compounds relies on the compounds being present in the available compound libraries. The libraries employed in this study were not developed specifically for this application and hence some compounds present at fire scenes may not be represented. These compounds were still included because they were present at high intensity and were therefore considered important. Although identification was not possible, the presence of these compounds at high intensity in casework samples could be valuable information to indicate that potentially hazardous compounds are being released by a fire. Ideally, all identifications made would be cross-checked with standards, but given the sheer number of compounds detected, this was outside the scope of this research.

**Figure 4.**
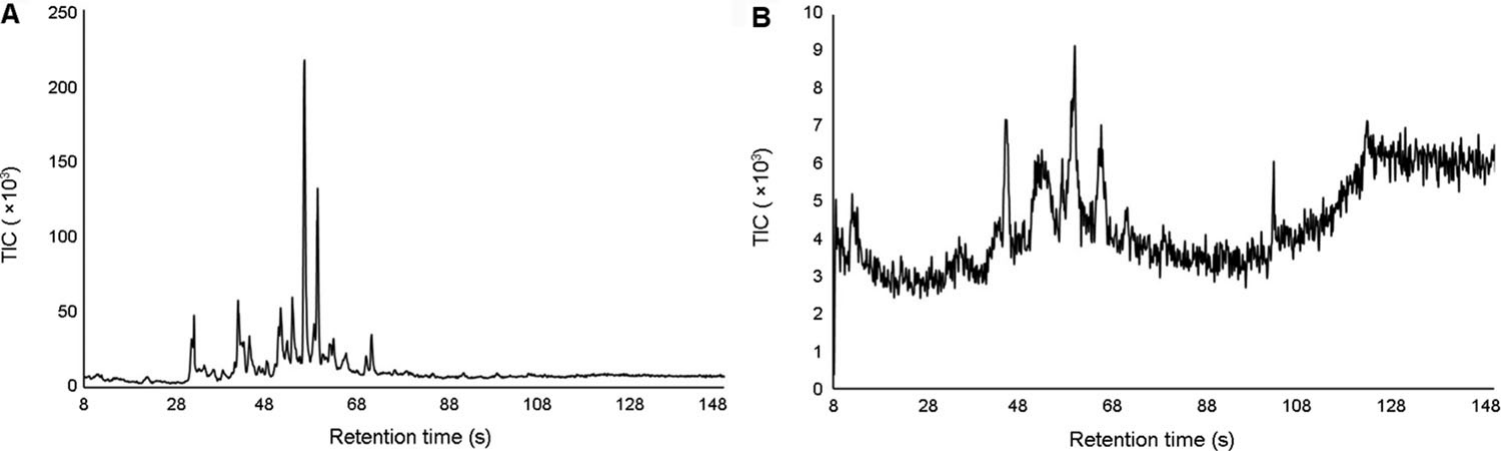
Representative background chromatograms of air samples collected from paper and cardboard burns 1 min after the fuel had self-extinguished. The air sample collected at this time point is reflective of the background that would be present during the individual material burns: (A) highest and (B) lowest background signal observed. TIC: total ion current.

Compounds detected in the background paper/cardboard burns (Figure 4) were present at a range of intensities, with isopropyltoluene being the most abundant. However, this compound was not detected during the combustion of the six materials of interest (Table 2). The other compounds detected in the paper/cardboard burns were detected during the material burns. Whilst the paper/cardboard background signal might contribute to the compound profile of the air samples collected during the combustion of the different materials, the greater signal intensity of these compounds in the material burns compared to the paper/cardboard burns suggest that these compounds are also likely formed by the combustion of the different materials.

A range of compounds were identified across all materials burned; these included benzene, toluene, ethylbenzene, xylenes, styrene and naphthalene. Not only were these compounds always present, but they were always present at high levels, relative to the other compounds present. It was also noted that different materials provide for different chemical profiles in the smoke plume during the fire. None of the materials are identical in the compounds that they release, indicating that the compound profile—as would be expected—will change depending on the fuels being burnt. Particle board, melamine and laminated wood emitted similar VOC/SVOC profiles, thus suggesting that these materials are of similar composition and confirms that the experimental parameters and field-based sampling and analysis provide for consistent results.

Further to the trends observed above, the results can also be validated by previous research into the release of hazardous compounds from the pyrolysis and combustion of common materials. Many compounds that were detected and identified using the field-based air sampling and analysis method have previously been identified using laboratory-based pyrolysis and combustion tests. Table 2 includes literature references where similar compounds were reported during the pyrolysis or combustion of similar fuel sources. Note that only the first iteration of each isomer has been referenced. These results demonstrated that similar identifications were obtained using the field-based sampling and analysis method as were reported in the literature from controlled laboratory experiments. The correlation between these two datasets reinforces the validity of the results obtained in the field using the proposed methodology.

### Release of VOCs and SVOCs after the fire

Post-extinguishment sampling and analysis results were also processed to determine if any VOCs and/or SVOCs are being released from fire debris even after water extinguishment. Compounds still emanating from the debris could pose a hazard to first responders, the environment and the general public. Any additional compounds identified post-extinguishment were added to the target list generated from air samples taken during the fire (Table 2). These combined results are presented in Table 3.

**Table 3.** Target compound list for in-field air sampling and analysis after extinguishment of a fire.

Compound name	PB	Melamine	LW	Carpet	Rubber	Underlay
Butanol					✔	✔
Dimethylpentane isomer						
Butadiene isomer					✔	✔
Methylbutane isomer						
Acrolein						
*Trimethylamine*	✔	✔			✔	
Butadiene isomer						
Acetone	✔	✔			✔	✔
Methylbutane isomer						
Acrylonitrile						
Methylbutadiene isomer					✔	✔
Furan	✔	✔			✔	
Pentenyne isomer		✔				✔
Methylpentane isomer						✔
Methylpentane isomer						
Dihydrofuran isomer			✔			
Butanone isomer						
Hexane						✔
Methylfuran isomer		✔				✔
Acetic acid	✔		✔			
Methyloctene isomer						
Cyclohexadiene				✔	✔	
Acetic anhydride	✔					
Benzene	✔	✔	✔	✔	✔	✔
Heptane						
*Unknown 1*	✔		✔			
*Dimethylcyclopentane*						✔
Ethyldimethylpentane isomer						✔
Dimethylfuran isomer						
*Unknown 2*		✔				
Methylpyrrole isomer	✔	✔				
Methylpyrrole isomer		✔				
*Unknown 3*					✔	
Hexynone isomer	✔	✔	✔			✔
Pyridine	✔					
Unknown 4	✔					
*Unknown 5*		✔	✔			
*Unknown 6*						✔
Unknown 7	✔					
*Unknown 8*			✔			
Toluene	✔	✔	✔	✔	✔	✔
Methyleneheptane isomer				✔	✔	
*Hexanal* [22]						✔
Octene isomer				✔	✔	✔
*Unknown 9*						
Unknown 10						
Octene isomer				✔		
Unknown 11						
Furfural		✔				
Furfural	✔	✔	✔	✔	✔	
Methylfuran isomer	✔	✔	✔			
Methyloctane isomer					✔	
Furanmethanol isomer	✔	✔	✔			✔
Furanmethanol isomer	✔	✔	✔		✔	
Ethylbenzene		✔	✔		✔	✔
Unknown 12						
*Unknown 13*	✔					
Xylene		✔			✔	✔
*Unknown 14*		✔				
*Unknown 15*	✔	✔				
Phenylethyne			✔		✔	✔
Heptanone			✔			✔
*Cyclohexanone isomer* [26]	✔	✔	✔		✔	✔
*Unknown 16*						✔
Styrene	✔	✔	✔	✔	✔	✔
Xylene		✔	✔		✔	✔
Cyclohexanone	✔				✔	✔
Unknown 17						
*Unknown 18*						✔
Unknown 19						
*Methylcyclopentanone isomer*	✔	✔	✔		✔	✔
*Furanylethanone isomer*		✔	✔		✔	✔
Furanone isomer	✔	✔	✔		✔	
ɑ-Pinene	✔	✔				
*Unknown 20*	✔					
*Unknown 21*		✔				
*Chloropropanediol isomer*		✔	✔			
Ethylhexanal isomer	✔			✔		
Camphene	✔				✔	✔
Propylbenzene isomer						✔
Methylfurancarboxaldehyde isomer	✔	✔	✔		✔	
Trimethylbenzene isomer		✔	✔		✔	✔
*Chlorotoluene*		✔				✔
Dimethylpyrrole isomer	✔	✔	✔		✔	
Phenol	✔		✔			
Aniline		✔			✔	
ɑ-Methylstyrene	✔	✔			✔	✔
Trimethylbenzene isomer					✔	✔
*tert Butyl benzene*					✔	
Benzonitrile	✔	✔			✔	✔
Indane		✔			✔	✔
Dimethylpyrrole isomer	✔					✔
Benzaldehyde	✔	✔		✔	✔	✔
Unknown 22						
Trimethylbenzene isomer		✔			✔	✔
Benzofuran		✔				
Methylethylheptane isomer	✔	✔	✔	✔	✔	✔
Ethylhexanol isomer		✔		✔		
*Unknown 23*	✔					
*Unknown 24*						
*Unknown 25*		✔	✔			
β-Pinene					✔	✔
Limonene	✔	✔	✔		✔	✔
*Hydroxymethylcyclopentenone isomer*	✔	✔	✔			
*Isopropyltoluene*					✔	✔
Unknown 26						
Methylphenol isomer	✔	✔	✔		✔	✔
Unknown 27	✔					
Indene	✔	✔	✔	✔	✔	✔
m-Cresol						
Methylphenol isomer	✔	✔	✔		✔	
Acetophenone		✔	✔			
Methoxyphenol isomer	✔	✔	✔		✔	✔
Methoxyphenol isomer	✔	✔				
*Methylbenzaldehyde isomer*		✔			✔	✔
Unknown 28	✔					
*Nonanal* [22]		✔			✔	
Tridecane						
*Dimethylphenol isomer*	✔	✔	✔		✔	✔
Unknown 29						
Unknown 30						
*Dimethylphenol isomer*		✔	✔		✔	✔
*Dimethylphenol isomer*	✔		✔		✔	
*Unknown 31*					✔	✔
*Unknown 32*						✔
Methylindene isomer		✔		✔	✔	✔
Methoxymethylphenol isomer	✔		✔			
Methylindene isomer		✔	✔		✔	✔
Methoxymethylphenol isomer	✔	✔	✔		✔	
Naphthalene	✔	✔	✔	✔	✔	✔
Benzothiophene					✔	✔
*Benzothiazole*					✔	
Ethylmethoxyphenol isomer	✔	✔	✔		✔	
Hydroxylmethylacetophenone isomer	✔	✔	✔		✔	
Methylnaphthalene isomer	✔	✔			✔	✔
Methylnaphthalene isomer	✔				✔	✔
Eugenol	✔	✔	✔			
*Unknown 33*			✔			
Unknown 34						
Unknown 35						
Biphenyl		✔	✔		✔	✔
Tetradecane	✔	✔			✔	✔
Eugenol	✔	✔	✔			
Eugenol	✔	✔	✔			
Acenaphthene		✔			✔	✔
Acenaphthylene	✔		✔		✔	✔
Ethoxymethylmethoxyphenol isomer						
Unknown 36						
Unknown 37						
Fluorene			✔		✔	
Anthracene					✔	
*Phenanthrene* [18,19,22,27,28,30,32,33]					✔	
*Fluoranthene* [18,19,22,30,32,33]					✔	
*Pyrene [*18,19,22,30,32,33]					✔	

Compounds identified after extinguishment but not during the fire are indicated in italics. In-text citations are included for compounds previously identified within published literature searches of by-products of pyrolysis and combustion. ✔: compound detected after extinguishment; PB: particle board; LW: laminated wood. Note: Only the first iteration of each compound was referenced.

The results obtained after extinguishment were interesting in that the range of compounds detected were primarily SVOCs and later-eluting VOCs. In particular, the highly volatile compounds were either not detected at all, or were present at levels lower than typically detected when sampling during the fire. It is likely that these more-volatile compounds have been consumed during the fire and hence are not present, or only present in low levels post-extinguishment.

A range of compounds were also detected in the air samples collected from the fire debris that were not detected in air samples collected during the fire. These are indicated in italics in Table 3. Additional compounds detected post-extinguishment could be attributed to inefficient smouldering combustion that occurs after extinguishment of the fuel source. During active combustion, it is likely that these compounds are consumed or broken down within the fire and hence are not detected in the air sample collected during the fire. Smouldering combustion could also account for the increased abundance of large volatile and semi-volatile organic compounds.

Interestingly, the compounds that are always detected in air samples collected during a fire (benzene, toluene, ethylbenzene, xylenes, styrene and naphthalene) were not all detected after extinguishment. Benzene, toluene, styrene and naphthalene were still present in all the samples, whilst the other compounds were detected in the fire debris from some materials but not others.

The overall results for post-extinguishment were consistent across the materials burnt, similarly to the results obtained for during the fire. There is good correlation between the laminated wood, melamine and particle board as previously found with the air samples collected during the fire. Rubber and underlay also show similarities in the VOC/SVOC profiles, whilst being different from the wood-based materials. The carpet debris released similar compounds to the rubber and underlay.

Despite there being no active smoke plume or other visual cues that hazardous compounds are present, the results obtained during this project have shown that, even after the fire has been extinguished, there is still significant off-gassing occurring within the debris. These hazardous organics pose a threat to first responders who remain at the scene of a fire after extinguishment. Although the presence of these hazardous organics will decrease over time, it is important to recognise that an extinguished fire still presents a toxic and hazardous environment, and any active personnel attending the scene should take precautions to prevent exposure.

### Benchmarking against conventional sampling and laboratory analysis

**Figure 5.**
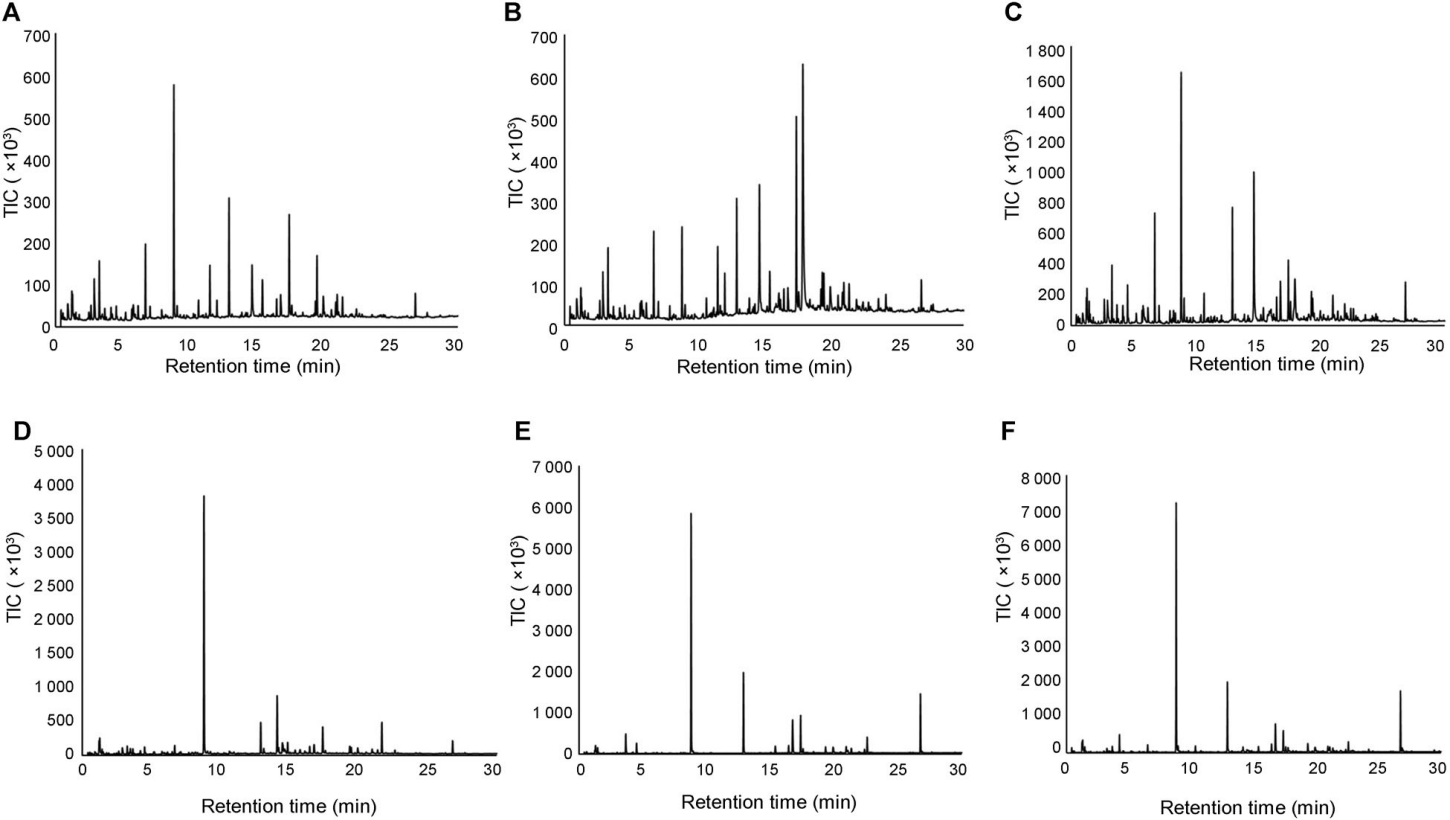
Representative chromatograms generated using an air analysis system of air samples collected during material burns: (A) particle board, (B) melamine coated particle board, (C) laminated wood, (D) carpet, (E) rubber and (F) underlay. TIC: total ion current.

The results obtained using the field-based method were compared to the results provided by conventional air sampling and laboratory-based analysis techniques. Figure 5 shows the results obtained from the air canister samples collected for each material that was burned.

When comparing the portable GC-MS results with the laboratory-based air analysis results, it was apparent that the portable GC-MS was not as sensitive as the laboratory-based method. This is not unexpected, given that the laboratory air analysis system is a dedicated system that can only be used for air analysis, whilst the portable GC-MS is designed as a generic tool to be used for a range of applications. The laboratory air analysis system utilises a liquid nitrogen cryotrap method to trap the compounds present in an air sample prior to GC-MS analysis. Whilst cryotrapping provides very high sensitivity, it is not a feasible method for field use.

The rationale for using the person-portable GC-MS for this application must be considered in context. The primary aim of the person-portable GC-MS is to provide more information than is currently available on the release of hazardous compounds at fire scenes, whilst the fire is burning, to aid in rapid site risk assessment and management. The aim of the portable GC-MS is also to trigger an emergency response, and alert first responders that they might be dealing with a potentially hazardous situation. The portable GC-MS has been demonstrated to achieve this aim despite not being as sensitive as traditional laboratory-based methods.

In addition, although the portable method is not as sensitive as the conventional laboratory-based method, there is a significant advantage associated with the use of the NT sampling device in conjunction with the portable GC-MS when compared to the laboratory-based air analysis system. The laboratory-based system is capable of analysing compounds with a boiling point of up to around 200˚C–250˚C. Compounds with a higher boiling point are not released into the GC-MS for analysis. Methyl-naphthalenes (or compounds around a similar boiling point) are the largest compounds able to be detected on the air analysis instrument. Although this is suitable for general air analysis, the results obtained using the portable GC-MS indicate that this is not always suitable for samples collected from fire scenes.

The NT device traps a range of compounds on a sorbent bed including both VOCs and SVOCs. All compounds that are trapped on the sorbent bed are released into the GC-MS, meaning that SVOCs with boiling points over 200˚C–250˚C can be captured and analysed. Many SVOCs were detected in the field-based results (Table 3). These are mostly heavier polyaromatic hydrocarbons (PAHs), indicating that exposure to heavy PAHs through air is a possibility at fire scenes. Although the portable GC-MS is more generic in nature than the laboratory air analysis system and therefore not as sensitive, it does mean that the field methods can detect a wider range of compounds. Given the nature of field-based methods as preliminary screening tools, this is highly advantageous.

The results from the air samples analysed using the conventional laboratory-based method were processed and the more prominent peaks present in the chromatograms identified. It was decided that only the higher intensity peaks within the laboratory-based results would be compared with the field-based data. If the portable GC-MS is capable of determining the presence of the higher-concentration compounds, it would trigger an emergency response and further laboratory testing. The field-based methods would then be deemed operationally suitable. Conversely, if these larger peaks are not detected at a particular fire scene, it would be considered unlikely that the lower-concentration compounds are also present and further investigation is not likely required.

To explain this with an example, Figure 5(E) illustrates the results obtained for rubber. There are 19 significant peaks present and, of these peaks, 16 stand out in terms of intensity. The total number of peaks detected in this sample was around 50, but the majority of the peaks were at low concentration (not visible in Figure 5(E) due to the low intensity of these peaks compared to the larger intensity compounds). Although some of these might be detected by the portable GC-MS, it is unlikely that all of these compounds would be detected. If the portable GC-MS was able to detect the 19 higher intensity peaks detected by the laboratory-based air analysis system, it was deemed suitable to the application. Therefore, the identity of the 19 higher intensity peaks detected by the laboratory technique was determined based on library searches using the software on the air analysis instrument. The 19 larger peaks were included in Table 4. This general approach was applied to all the other materials burned and the detected compounds were compared to those detected by the portable GC-MS for the same materials burned.

**Table 4.** Compound identifications obtained from air samples collected from small-scale burns using a canister followed by laboratory-based analysis.

Compound name	PB	Melamine	LW	Carpet	Rubber	Underlay
Acrolein	✔	✔	✔	✔		✔
Butadiene isomer	✔		✔	✔	✔	✔
Butenyne isomer [19,20]			✔	✔	✔	✔
Unknown 1	✔	✔	✔	✔		
Unknown 2	✔	✔	✔	✔		
1H-imidazole	✔	✔	✔	✔		✔
Unknown 3				✔		
Methylbutadiene isomer	✔	✔	✔	✔	✔	✔
Methylacetic acid ester	✔	✔	✔			
Bicyclo[2.1.0]pentane				✔		
Pentenyne isomer	✔	✔	✔	✔	✔	
Dihydrofuran isomer	✔	✔	✔			
Butynol isomer		✔	✔			
Methylbutene isomer	✔	✔	✔			
cis-Cyclohexanediamine isomer		✔				
Methylhexanone isomer	✔	✔	✔			
Butene						✔
Methylfuran isomer	✔	✔	✔	✔		
Methylnitropropane isomer						
Methylfuran isomer	✔	✔	✔			
Dimethylcyanamide isomer	✔	✔	✔			
Methylcyclopentadiene isomer			✔			
Methylcyclopentadiene isomer			✔			
Benzene	✔	✔	✔	✔	✔	✔
Thiophene					✔	✔
Methylpentene isomer [17]	✔	✔	✔			
Dimethylhexene isomer						✔
Dimethylfuran isomer	✔	✔	✔			
Methylpyrrole isomer	✔	✔				
Pyrrole [19,22]	✔	✔				
Toluene	✔	✔	✔	✔	✔	✔
Ethylacrolein				✔		
Pyridinone isomer		✔				
Methylbutene isomer				✔		✔
Methylheptene isomer				✔		
Isopropylcyclobutane						✔
Ethylmethylpentene isomer				✔		
Pyridinone	✔	✔	✔			
Chlorobenzene [21, 29]				✔		
Furanmethanol		✔				
Ethylbenzene	✔	✔	✔	✔	✔	✔
Xylene	✔	✔	✔	✔	✔	✔
Styrene	✔	✔	✔	✔	✔	✔
Xylene	✔		✔	✔	✔	✔
Methylcycloheptane isomer						✔
Methylfurancarboxaldehyde isomer		✔	✔			
Formyloxyphenylethanone isomer	✔	✔	✔	✔	✔	✔
Methylnitropropane isomer				✔		
ɑ-pinene	✔	✔	✔			
Benzonitrile	✔	✔	✔	✔	✔	✔
ɑ-Methylstyrene	✔	✔				
β-Pinene	✔	✔				
Benzofuran	✔	✔	✔	✔	✔	✔
Trimethylbenzene isomer		✔	✔		✔	✔
Ethylhexanol isomer				✔		
Unknown 4		✔	✔			
Limonene		✔	✔		✔	✔
Indene			✔	✔	✔	
Formyloxyphenylethanone isomer		✔				
Methoxyphenol isomer		✔	✔			
Dodecenol		✔				
Naphthalene	✔	✔	✔	✔	✔	✔
Benzothiophene						✔

In-text citations are included for compounds previously identified within published literature searches of by-products of pyrolysis and combustion. PB: particle board; LW: laminated wood; ✔: compound detected; ✔: compound also detected in same sample using the portable GC-MS in the field. Note: Only the first iteration of each compound was referenced.

The previous issue with compound identifications on the portable GC-MS was also encountered on the air analysis system when attempting to identify peaks present using library searching. The NIST library was used for compound identifications and this approach could only identify some compounds with certainty, whilst other compounds could only be tentatively identified or not matched at all. These latter compounds have been indicated in the results as “unknown”.

All the major compounds detected by the laboratory-based method were also detected using the field-based method. The high correlation between these two lists of compounds highlights the validity of the results obtained using the portable GC-MS. Due to the similarities between the field and laboratory results, it is possible to cross-check and validate the presence of the hazardous organic materials using the conventional laboratory-based analysis technique, thereby providing confirmation of the in-field results. Likewise, some of the compounds have been identified previously in the literature, providing an external validation of the field- and laboratory-based results.

The compounds identified from the controlled burns were also compared to air monitoring target lists of known toxicants. Air monitoring protocols for the determination of toxic organic compounds in ambient air—such as EPA Method TO-14 [34] and TO-15 [10]—target specific hazardous compounds that pose risk to human and environmental health. Ordinarily, when these procedures are undertaken, only the target compounds of these methods are reported. Any other compounds present in the samples are not reported. Many of the compounds that were observed to be present during and after the fire are not part of the target TO-14/15 lists and, as such, are not normally reported when these analyses are requested on samples from fire scenes. This observation highlights the need for the development of a fire-specific target library. Through the development of a list of fire-specific target compounds, it becomes easier to identify the range of compounds that are potentially released during a fire. This can better-inform policies and procedures surrounding environmental and human health protection, and can assist in the generation of toxicity data for fire-related toxicants. The results presented here form the basis of such a list and, through further research and testing, the identification of many of the unknown peaks reported here could be achieved.

### Implementation at fire scenes

The primary aim of person-portable instrumentation is to provide more information to first responders on the release and identification of pollutants at fire scenes than is available using current laboratory-based approaches. The field-based GC-MS method was capable of providing consistent and timely results in a controlled field setting. VOCs and SVOCs were successfully identified whilst an active fire was on-going and also immediately after extinguishment.

The turnaround for field-based air sampling and analysis was approximately 10 min (accounting for sampling time, analysis time and an NT/system cleaning step). This has the added benefit of being able to provide contemporary intelligence, giving that continuous sampling and analysis is possible throughout the duration of the fire. It is possible to obtain continuous results as the fire develops and different volatiles are released due to different fuel sources being consumed. Information of this nature is not currently available to first responders during an active emergency such as a large factory fire.

The NT was able to be easily utilised for simple and effective extraction of VOCs and SVOCs from air. It is possible to integrate the field-based sampling and analysis methods developed here within a fire scene assessment protocol with minimal additional training for first responders. The results obtained using these methods could thus be utilised within the emergency response protocol for more efficient protection strategies and sample triaging.

The NT system is light and small and can be clipped to a fire fighter’s suit, allowing the fire fighter to collect samples whilst still fighting the fire or conducting other duties related to the fire scene. The collected NT sample is then analysed on the portable GC-MS either by the fire fighter themselves, or the NT can be handed over to an analyst in the cold/safe zone of the scene for subsequent analysis. Training on the use of NT is straightforward and can be performed in a few minutes. The training on the portable GC-MS is made easy by the on-screen visual prompts guiding the user in correct injection procedure.

The data processing is conducted automatically by the portable GC-MS as part of its standard analysis procedure. The peaks present within the chromatograms are automatically de-convoluted, detected and identified using the target and on-board libraries. These compounds are then tabulated and can be viewed on the portable GC-MS. Through continual use and refinement, the library will become increasingly customised to organic compounds released from a fire.

Additionally, the results obtained provide a comprehensive screen of a range of volatile organics present in air. These results provide first responders with a quick indication of the composition of the air samples for initial environmental and human health monitoring. Compounds that are detected and identified using the field-based method can be confirmed using laboratory-based air sampling and analysis methods, and as such, the developed field-based method can be easily incorporated into the current sampling and analysis protocols.

## Conclusion

The research presented here confirmed that person-portable instrumentation has the ability to provide on-going support to first responders in emergency scenarios. Specifically, the application of a person-portable GC-MS for real-time air sampling and analysis at fire scenes can provide vital knowledge to first responders regarding the release of hazardous organic compounds into the atmosphere that pose risks to human and environmental health. The field-based methods employed in this study have been demonstrated to provide consistent results directly related to the materials burnt. The results were validated using laboratory-based air sampling and analysis procedures on air samples taken from the same fires as well as previously-published research into the pyrolysis and combustion of similar materials. The field-based methods were able to detect and identify a greater range of VOCs/SVOCs than conventional laboratory-based sampling and analysis methods, and demonstrated a capability to be used as an effective screening tool for air monitoring at fire scenes. It was demonstrated that, through the employment of these rapid, field-based air sampling and analysis methods, it is possible to provide first responders with the necessary contemporary intelligence on the release of hazardous organic compounds at active fire scenes. This can facilitate proactive and targeted risk assessment and management for the protection of human and environmental health.
